# Association between PIK3CA alteration and prognosis of gastric cancer patients: a meta-analysis

**DOI:** 10.18632/oncotarget.23871

**Published:** 2018-01-02

**Authors:** Hua Li, Shubo Chen, Hui Li, Jianxin Cui, Yunhe Gao, Dianchao Wu, Shangfeng Luan, Yan Qin, Tongshan Zhai, Dengxiang Liu, Zhibin Huo

**Affiliations:** ^1^Department of Surgical Oncology, Affiliated Xing Tai People Hospital of Hebei Medial University, Xingtai 054001, China; ^2^Department of Surgical Urology, Affiliated Xing Tai People Hospital of Hebei Medial University, Xingtai 054001, China; ^3^Department of General Surgery, Affiliated Xing Tai People Hospital of Hebei Medial University, Xingtai 054001, China; ^4^Department of General Surgery, Chinese People’s Liberation Army General Hospital, Beijing 100853, China; ^5^Institute of Cancer Control, Affiliated Xing Tai People Hospital of Hebei Medial University, Xingtai 054001, China

**Keywords:** PIK3CA, gastric cancer, prognosis, alteration, meta-analysis

## Abstract

**Background:**

Increasing evidence suggests that dysregulation of phosphatidylinositol-4, 5-bisphosphate 3-kinase, catalytic subunit alpha (PIK3CA) plays an important role in carcinogenesis. However, the relationship between PIK3CA expression and gastric cancer (GC) prognosis remains controversial.

**Methods:**

We searchedPubMed, Embase, Web of Science, and the Cochrane Library databases for relevant studies up to June 30, 2017. Primary outcomes were hazard ratio (HR), odds ratio (OR), and 95% confidence intervals (CI) for association with overall survival and clinicopathological features.

**Results:**

Eleven studies comprising 2481 GC patients were analyzed. Pooled analysis showed that PIK3CA upregulation was significantly associated with worse overall survival (HR = 1.79, 95% CI 1.42–2.27, *p*< 0.001) at the protein (HR = 1.94, 95% CI 1.52–2.47, *p*< 0.001) but not the gene (HR = 1.57, 95% CI 0.92–2.69, *p*= 0.097) level. *PIK3CA* gene mutation did not correlate with overall survival (HR = 1.05, 95% CI 0.83–1.34, *p*= 0.666) but was significantly associated with poor tumor differentiation (OR = 0.37, 95% CI 0.17–0.76, *p*= 0.011).

**Conclusion:**

High PIK3CA protein expression predicted poor prognosis in GC, whereas *PIK3CA* gene amplification or mutation did not. Moreover, *PIK3CA* mutation was an indicator of poorly differentiated tumors.

## INTRODUCTION

Gastric cancer (GC) is the fourth most common malignant neoplasm worldwide, and it was estimated to be the second leading cause of cancer-related death in 2014 [1]. Despite recent breakthroughs in the diagnosis and treatment of GC, its prognosis remains unfavorable [2]. Therefore, understanding the biological alterations associated with GC, especially those leading to the dysregulation of signaling pathways, might help to predict patient prognosis and identify novel therapeutic targets.

The phosphatidylinositol 3-kinase (PI3K) pathway is one of the most commonly activated and altered signaling pathways in cancer, including GC [3–5]. Since the PI3K pathway plays an essential role in several cellular processes, including cell growth, metabolism, and survival, it is not surprising that its dysregulation is also of critical importance to pathological processes such as the development, progression, and metastasis of cancer [6, 7]. A key step in the PI3K/AKT pathway is the generation of phosphatidylinositol-3, 4, 5-trisphosphate (PIP3) by PI3K. The PI3K family exists as three subfamilies, one of which is composed of a p110 catalytic subunit coupled to a regulatory subunit. p100α, the gene product of phosphatidylinositol-4, 5-bisphosphate3-kinase, catalytic subunit alpha (*PIK3CA*), is of particular importance in signaling through the canonical PI3K pathway [8, 9]. Recently, large-scale next-generation sequencing studies have identified mutations in *PIK3CA* in Epstein–Barr virus (EBV)-associated and microsatellite instability (MSI)-associated GCs [10]. However, the prognostic value of PIK3CA alterations at both the protein and transcriptional levels in GC patients remains unclear and controversial.

Therefore, we conducted a meta-analysis of published studies to elucidate the precise relationship between PIK3CA dysregulation and the prognosis and other clinicopathological features of GC patients.

## RESULTS

### Eligible studies and general characteristics

**Figure 1:**
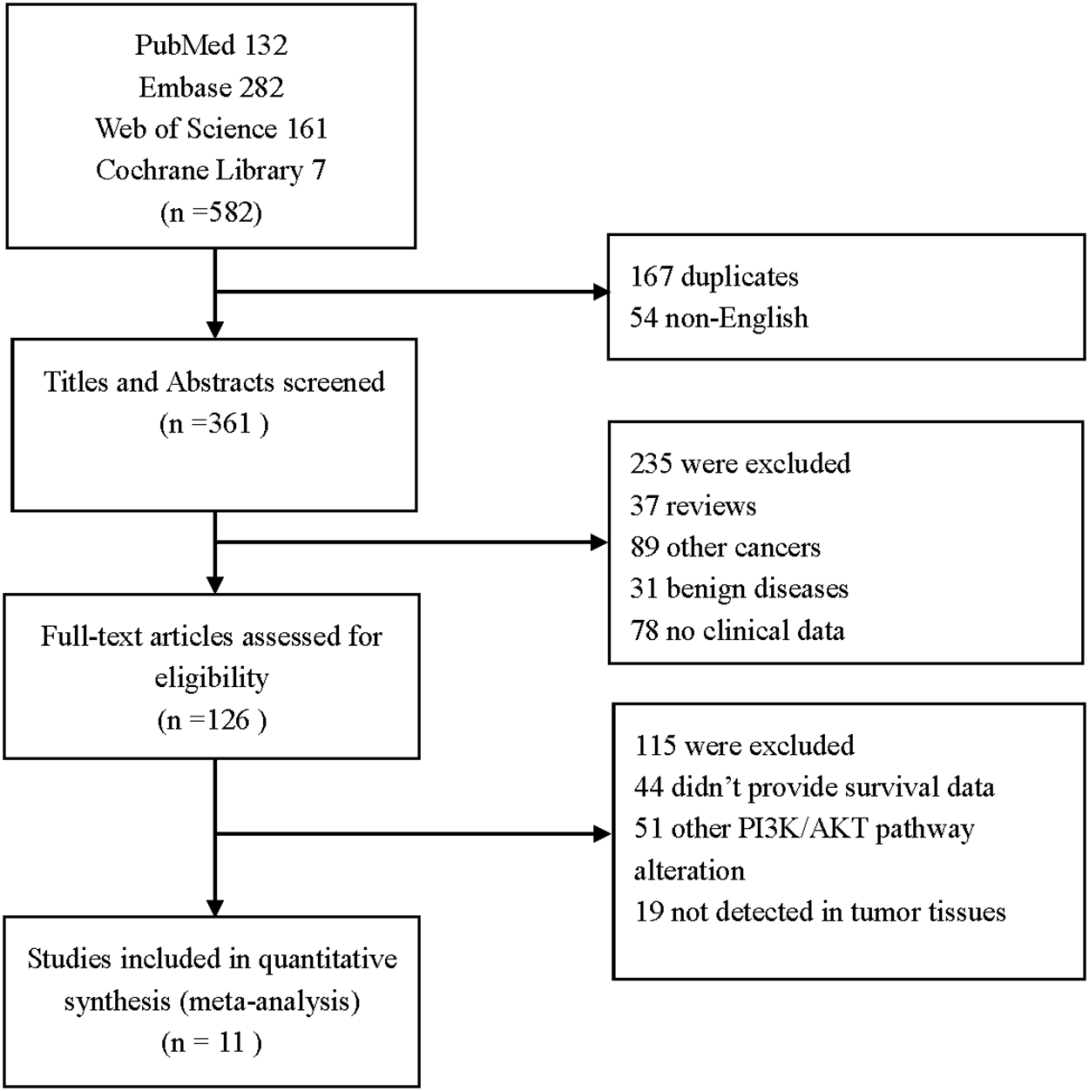
Flow chart of study selection process

A total of 582 studies were retrieved from a search of PubMed, Embase, Web of Science, and Cochrane Library online databases using established strategies (see Materials and Methods). After a review of the titles and abstracts, 167 duplicates and 54 non-English language studies were excluded. Of the remaining 361 studies, 235 were excluded because they did not meet the eligibility criteria of our analysis. Another 44 studies did not report patient survival, 51 focused on components of the PI3K/AKT pathway other than PIK3CA, and 19 did not examine PIK3CA expression in cancer tissues. Finally, a total of 11 studies were included in our meta-analysis (Figure 1).

The general characteristics of the included studies are presented in Table 1. The 11 studies comprised 2481 patients for pooling analysis (median 208, range 79–568). Two studies were from Europe (UK and Italy) and the remaining nine were from East Asia (four from China, two from Japan, two from Korea, and one from Singapore). The various techniques used for the qualitative and quantitative detection of PIK3CA included polymerase chain reaction (PCR) followed by direct sequencing or pyrosequencing of amplicons, quantitative real-time PCR (qPCR), and immunohistochemistry (IHC) to detect protein. All studies were scored above 5 stars except Y Sukawa’s and M Liang’s reports according the Newcastle–Ottawa Scale (NOS) [22], which indicated a relatively high risk of bias [23, 24] (Supplementary Table 1).

**Table 1:** Major characteristics of the selected studies

No.	First author	Year	Number	Sex(M/F)	Age	Region	TNM Stage^a^	Alteration type	Detection method	Treatment	Follow up	HR estimate	NOS score
1	S Barbi [11]	2010	264	175/89	67.4(median)	Italy	I-IV	Gene mutation(exon 9,20)	Direct sequencing	R0 Only	NR	Provided(M)	7
2	J Shi [12]	2012	131	102/29	NR	China	I-IV	Gene mutation(exon 9,20) and amplification	Direct sequencing and qPCR	R0/R1	NR	Provided(M)	7
3	Y Sukawa [13]	2012	231	157/74	71(25-91)	Japan	IB-IV	Gene mutation(exon 9,20)	Pyrosequence	NR	NR	FC	5
4	AFC Okines [14]	2013	337(303)^b^	263/74	63(median)	UK	I-IIIC	Gene mutation(exon 9,20)	Direct sequencing	R0 Only/R0+Chemotherapy	NR	FC	9
5	ML Chong [15]	2013	79	64/15	53% (≥69)	Singapore	I-IV	Gene amplification	qPCR	R0 Only	15(1-131)	Provided(U)	8
6	H Lee [16]	2015	110	83/27	50% (≥60)	Korea	I-III	Gene mutation (exon9,20) and Amplification	Direct sequence and qPCR	R0 Only	82.2(3.7-158.8)	FC	7
7	M Liang [17]	2015	107	NR	NR	China	I-IV	Protein overexpression	IHC	R0/R1	NR	FC	5
8	K Harada [18]	2016	208	148/60	NR	Japan	I-IV	Gene mutation(exon 9,20)	Pyrosequencing	R0 Only	60	FC	7
9	M Dong [19]	2016	568	396/172	52.3% (≥60)	China	I-IV	Protein overexpression	IHC	R0/R1	37.2(0-93.8)	FC	7
10	SH Jang [20]	2016	178	127/51	68%(≥55)	Korea	I-IV	Protein overexpression	IHC	R0+Chemotherapy	58(0-95)	Provided(M)	7
11	JW Kim [21]	2017	302	202/100	20.2%(≥70)	Korea	I-IV	Gene mutation	qPCR(exon 1,4,7,9,20)	R0/R1	NR	FC	7

^a^American Joint Committee on Cancer 7^th^ edition TNM stage system. ^b^ The actual number of patients analyzing PIK3CA status and survival was 303. FC: figure calculation; HR: hazard ratio; IHC: immunohistochemistry; M/F: male to female ratio; M: multivariate analysis; NOS: Newcastle–Ottawa Scale; NR: not reported; qPCR: quantitative polymerase chain reaction; U: univariate analysis; R0: radical resection; R1: palliative surgery.

### Association between PIK3CA overexpression in GC tissue and patient prognosis

**Figure 2:**
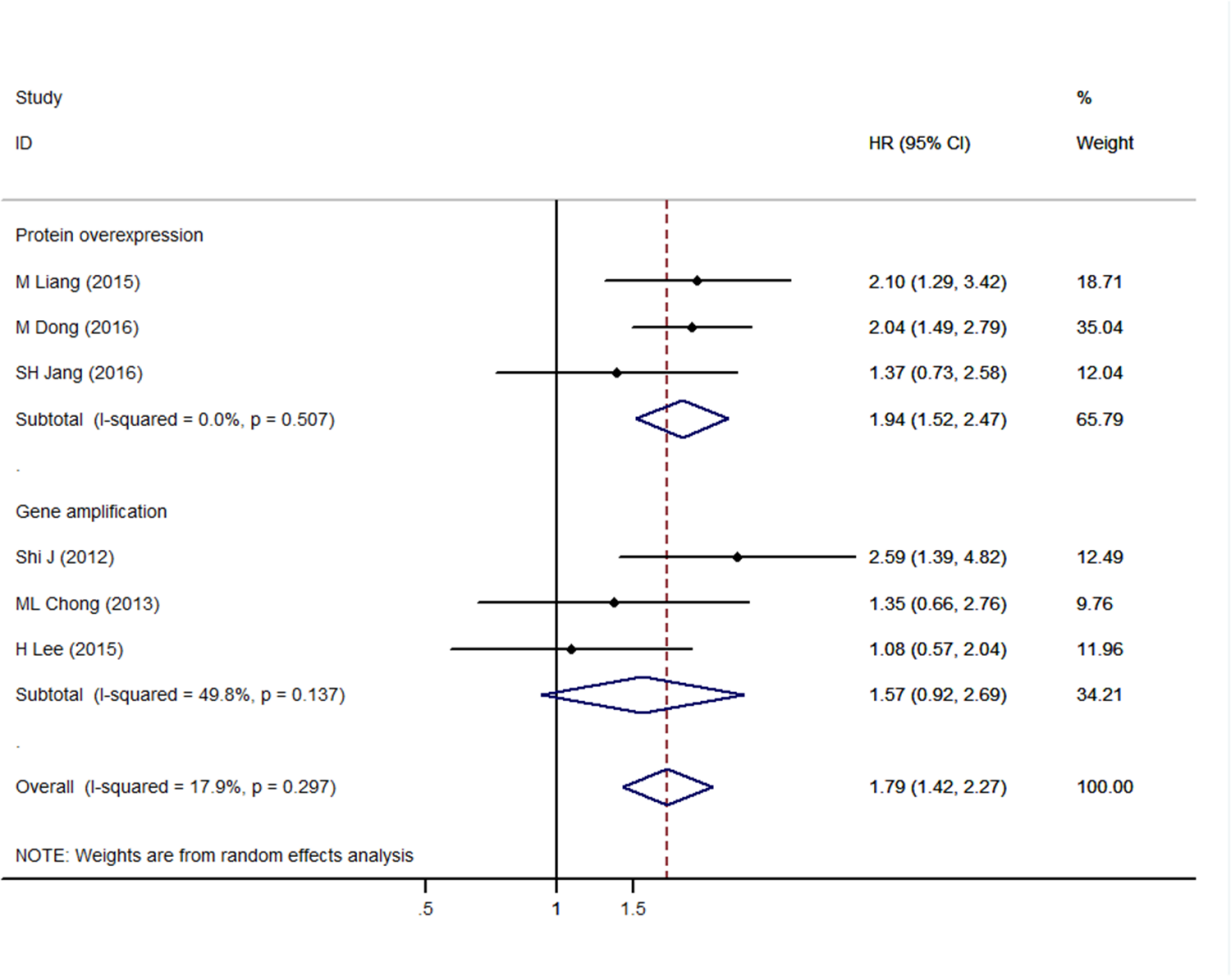
Forest plot of hazard ratios for overall survival of gastric cancer patients and PIK3CA overexpression stratified by PIK3CA alteration measurement

Six studies provided sufficient data to analyze the relationship between overall survival (OS) and PIK3CA expression level. The pooled analysis showed that high PIK3CA expression was associated with worse OS (hazard ratio [HR] =1.79, 95% confidence intervals [CI] 1.42–2.27, *p* < 0.001). Although no significant heterogeneity was observed (inconsistency index [*I*^*2*^] = 17.9%, *p*= 0.297), judging the presence of potential bias sources, a random-effects model was applied to make the conclusion more conservative and reliable [25] (Figure 2).

We then performed a subgroup analysis of studies that examined overexpression at the gene and protein level. The results showed that OS was significantly associated with PIK3CA protein overexpression (HR = 1.94, 95% CI 1.52–2.47, *p*< 0.001) but not *PIK3CA* gene amplification (HR = 1.57, 95% CI 0.92–2.69, *p*= 0.097) (Figure 2).

When it refers to the different treatment settings, subgroup analysis showed that in the radical resection and palliative surgery mixed group [12, 17, 19], the association between PIK3CA overexpression and overall survival remains significant (HR = 2.13, 95% CI 1.67–2.72, *p* < 0.001), while in the radical resection-only group [15, 16, 20], it was not significant (HR = 1.25, 95% CI 0.86-1.83, *p*=0.246), which might indicate a more predominant role of PIK3CA overexpression predication on survival in those patients with late-stage diseases.

Furthermore, the associations remained significant in both the univariate (HR=1.74, 95%CI 1.31-2.33, *p* < 0.001) and multivariate (HR=1.89, 95%CI 1.01-3.53, *p*=0.046) analysis models (Table 2, Left section).

**Table 2:** Subgroup analysis of PIK3CA status and patients’ survival

Subgroup	PIK3CA overexpression				*PIK3CA* mutation			
	Studies (Patients)	HR (95%CI)	Heterogeneity		Studies (Patients)	HR (95%CI)	Heterogeneity	
			I^2^ (%)	*P*			I^2^ (%)	*P*
Method								
IHC	3(853)	1.94(1.52-2.47)	0	0.507	/	/	/	/
qPCR and direct sequence	3(320)	1.57(0.92-2.69)	49.8	0.137	5(1110)	1.04(0.79-1.38)	0.0	0.840
Pyrosequence	/	/	/	/	2(439)	1.10(0.68-1.78)	0.0	0.476
Treatment								
R0	3(367)	1.25(0.86-1.83)	0	0.850	4(885)	0.99(0.71-1.38)	0.0	0.889
R0/R1	3(806)	2.13(1.67-2.72)	0	0.796	2(433)	1.07(0.69-1.65)	0.0	0.351
NR	/	/	/	/	1(231)	1.27(0.68-2.37)	/	/
Race								
Mongolian	6(1173)	1.79(1.42-2.27)	17.9	0.297	5(982)	1.08(0.79-1.47)	0	0.845
Caucasian	/	/	/	/	2(567)	1.02(0.69-1.51)	0	0.466
Analysis method								
Multivariate	2(309)	1.89(1.01-3.53)	49.4	0.160	2(395)	1.17(0.78-1.77)	0	0.453
Univariate	4(864)	1.74(1.31-2.33)	26.5	0.253	5(1154)	1.00(0.74-1.35)	0	0.906
HR evaluation								
Provided	3(388)	1.72(1.11-2.64)	23.1	0.272	2(395)	1.17(0.78-1.77)	0	0.453
Calculated	3(785)	1.80(1.28-2.53)	40.5	0.186	5(1154)	1.00(0.74-1.35)	0	0.906

HR: hazard ratio; R0: radical resection; R1: palliative surgery; NR: not reported; CI: confidence interval; I^2^: inconsistency index.

### Association between *PIK3CA* mutation in GC tissue and patient prognosis

**Figure 3:**
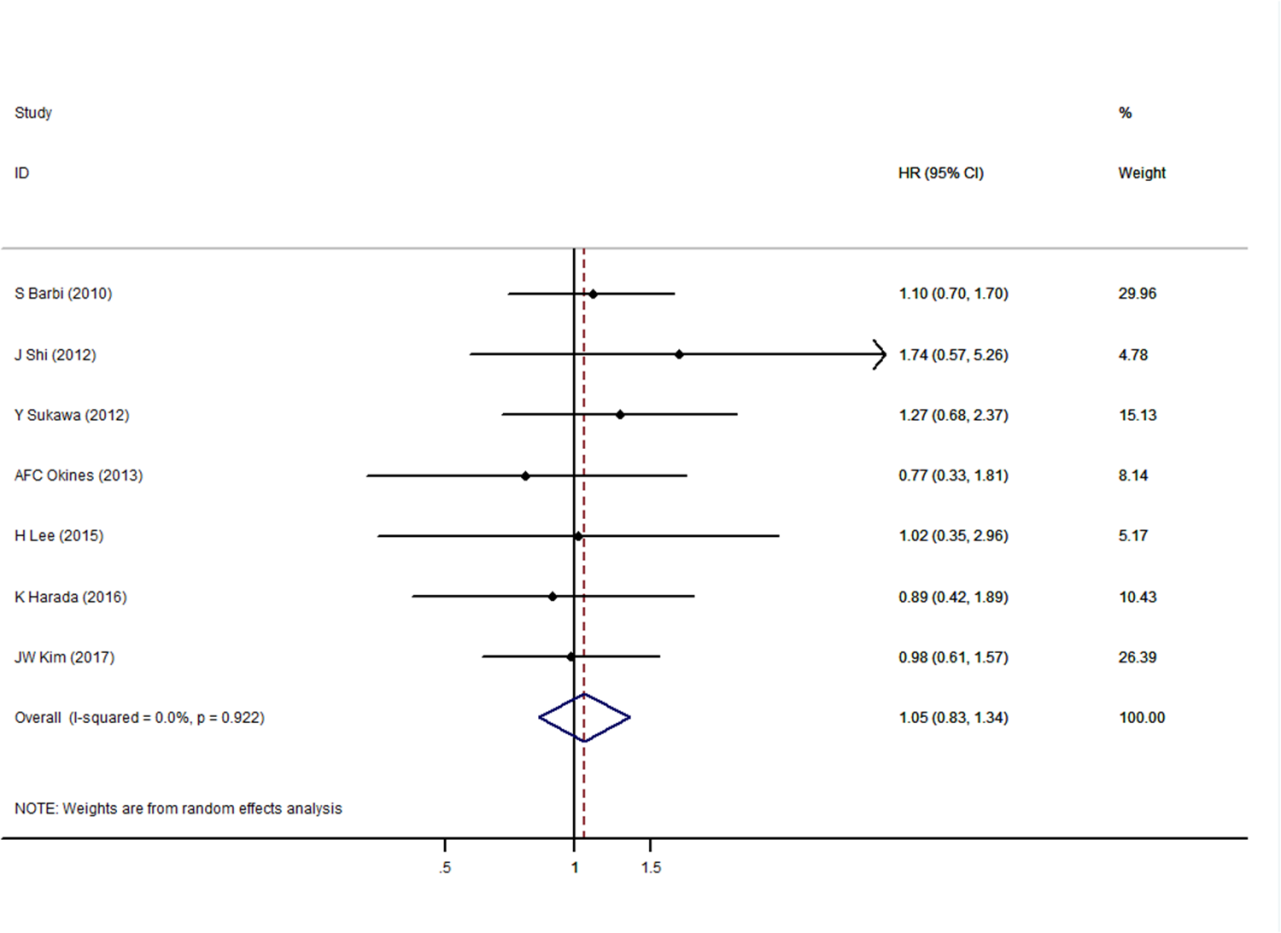
Forest plot of hazard ratios for overall survival of gastric cancer patients with *PIK3CA* gene mutations

A total of seven studies were pooled for the analysis of the effect of *PIK3CA* mutation on GC patient prognosis. The results showed that the presence of mutations (mainly in exons 9 and 20) did not correlate significantly with OS (HR = 1.05, 95% CI 0.83–1.34, *p* = 0.666) (Figure 3).

A subgroup analysis was also conducted due to the potential existence of study bias (Table 2, Right section). However, no obvious heterogeneity was observed and the association remains insignificant either in the subgroup of different statistical analysis or HR evaluation methods, which indicated a stable meta-analysis result within these subgroups.

### Association between PIK3CA dysregulation and clinicopathological features of GC patients

The associations between PIK3CA dysregulation (overexpression and mutation) and major clinicopathological features are shown in Table 3. Of the characteristics analyzed, the only significant association was between the presence of mutated *PIK3CA* and tumor differentiation grade (well/moderate vs. poor/undifferentiated, odds ratio [OR] 0.37, 95% CI 0.17–0.79, *p*= 0.011).

**Table 3:** Meta-analysis of the association between PIK3CA alterations and clinicopathological features of gastric cancer patients

PIK3CA overexpression Stratification	No. of studies	No. of patients	Pooled OR (95%CI)	Heterogeneity	
				I^2^(%)	*P*-value
Gender (M/F)	3	877	1.03 (0.77-1.39)	0	0.380
Lymph node metastasis (N0/N1-3)	3	877	2.23 (0.53-9.36)	91.1	<0.001
Distant metastasis (M1/M0)	2	1326	3.11 (0.35-27.42)	79	0.029
TNM stage (I+II/III+IV)^a^	3	877	0.52 (0.09-2.96)	95	<0.001
Differentiation (Well+Moderate/Poor)	2	308	1.31 (0.20-8.74)	88	0.004
Tumor size (<5cm vs ≥5cm)	2	699	4.43 (0.55-35.43)	85.8	0.008
Lauren classification (Intestinal/diffuse)	2	661	0.97 (0.16-6.04)	95.8	<0.001
**PIK3CA mutation Stratification**	**No. of studies**	**No. of patients**	**Pooled OR (95%CI)**	**Heterogeneity**	
				**I**^2^**(%)**	***P*-value**
Gender (M/F)	6	1200	0.93 (0.64-1.36)	0	0.822
Lymph node metastasis (N0/N1-3)	6	1183	0.95 (0.63-1.42)	0	0.762
TNM stage (I+II/III+IV)^a^	3	614	0.66 (0.39-1.12)	0	0.511
Differentiation (Well+Moderate/Poor)	2	386	0.37 (0.17-0.79)	0	0.522
Lauren classification (Intestinal/diffuse)	5	1009	0.76 (0.41-1.43)	52.7	0.076
Microsatellite instability (MSI/MSS)	2	566	0.68 (0.15-3.06)	82.9	0.016

^a^American Joint Committee on Cancer 7^th^ edition TNM stage system. CI: confidence interval; I^2^: inconsistency index; MSI: microsatellite instability.

MSS: microsatellite stability; OR: odds ratio.

### Publication bias

**Figure 4:**
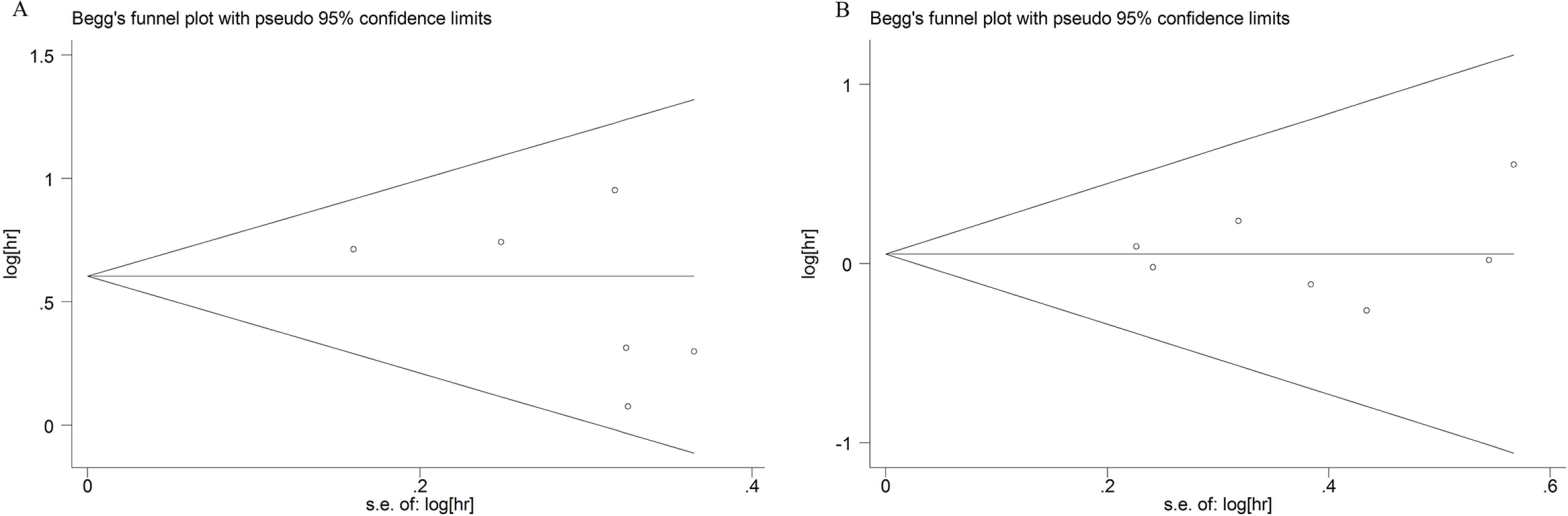
Funnel plot evaluation of potential publication bias on the effect of PIK3CA overexpression **(A)** and mutation **(B)** on overall survival of gastric cancer patients.

Potential publication bias was assessed in the association of OS with PIK3CA alteration type. As shown in Figure 4, no significant bias was detected in the included studies. For the overexpression studies, *p*= 0.260 for Begg’s test and *p*= 0.271 for Egger’s test, and for the mutation studies, *p*= 0.881 for Begg’s test and *p* = 0.22 for Egger’s test.

## DISCUSSION

The PI3K/AKT pathway is an important therapeutic target because its components, including PIK3CA, are frequently activated in cancer cells; indeed, several PI3K pathway inhibitors are currently in clinical trials [26, 27]. Therefore, identifying patients who might benefit from targeted therapy is of great importance [28, 29]. An increasing number of studies are focused on PIK3CA dysregulation and its potential impact in GC, one example being the MAGIC trial [30], highlighting the urgent need to elucidate more precisely the contribution of PIK3CA alterations to GC. To the best of our knowledge, this meta-analysis of 11 studies comprising 2481 GC patients represents the first comprehensive systematic evaluation of the prognostic value of PIK3CA alterations detected at the protein and gene levels. The results suggest that PIK3CA could represent a useful biomarker for predicting patient survival.

In theory, alterations in PIK3CA activity or expression could lead to downstream activation of PI3K/AKT pathway and promote the growth and invasion of GC cells [7, 31]. Our pooled analysis showed that PIK3CA dysregulation is associated with poorer tumor differentiation and worse OS among GC patients, indicating that PIK3CA is a prognostic biomarker for GC patients. However, the significant association between *PIK3CA* mutation and tumor differentiation should be interpreted with caution because of the small sample size (two studies comprising 386 patients) and need more studies to confirm. Furthermore, our results showed no significant association between *PIK3CA* mutational status and patient prognosis. This might be attributed to the fact that gene mutation of a single component does not necessarily reflect the status of an entire signaling pathway in terms of dysregulation and functional impact. Therefore, our results suggest that the detection of gene amplification and/or protein overexpression is a more direct and promising strategy to predict GC patient prognosis than gene mutation analysis.

The most recent NCCN guidelines for gastric cancer have described MSI and EBV status as potential molecular biomarkers for targeting treatment strategies in GC patients [32]. Although mutant *PIK3CA* is more common in MSI than in other GC types [10], our analysis failed to confirm this, possibly because of the small number of studies that analyzed MSI association (two studies comprising 566 patients). There were also insufficient data to analyze the association between *PIK3CA* mutation and EBV positivity, so further studies are necessary to evaluate this relationship.

Several limitations to our study should be noted. First, although we observed no obvious heterogeneity (Figures 2 and 3), the studies used different designs, PCR primers, and IHC antibodies, which might have contributed to heterogeneity. Therefore, a random-effects model was applied to make our results more conservative. Second, we obtained insufficient data to analyze the relationship between PIK3CA and disease-free survival, which reduces the predictive value of PIK3CA status on the outcomes of GC patients. Third, the included studies did not investigate the relationship between *PIK3CA* gene amplification and protein overexpression, which might be critical for selecting the appropriate method of analysis for different GC patients. Furthermore, association analysis of some clinicopathological features (Table 3) included only two to three studies, and therefore should be interpreted with caution; additional studies are required to confirm the conclusion. It is also noticeable that the patients from the MAGIC trial of AFC Okines’ study included a small portion of esophageal cancer patients (38 out of 337), which might add to the bias of our results. Finally, we limited the analysis to English language publications, which might have led to selection bias in our meta-analysis.

In conclusion, we found that overexpression of PIK3CA protein, but not the presence of *PIK3CA* gene amplification or mutations, predicted worse prognosis for GC patients. However, our findings, as well as other important variables such as the association of PIK3CA alterations with GC molecular subtypes, require further verification in more rigorous studies with consistent and standardized methodology.

## MATERIALS AND METHODS

### Literature search

The search was conducted by consulting PubMed, Embase, Web of Science, and Cochrane Library online databases from inception to June 30, 2017. The search terms were “gastric/stomach cancer” or “gastric/stomach neoplasm” and “PIK3CA” or “phosphatidylinositol-4, 5-bisphosphate 3-kinase, catalytic subunit alpha” and “prognosis” or “survival.” Article language was limited to English. The reference section of all relevant articles was examined to identify additional related studies. Two researchers (HL and YG) independently assessed the eligibility of the identified studies, and disagreements were resolved by discussion or consultation with a third researcher.

### Inclusion criteria

Studies were selected according to the following inclusion criteria: (1) inclusion of pathologically confirmed GC patients; (2) focus on PIK3CA alterations, at either the protein or gene level; and (3) inclusion of survival data according to the intra-tumor PIK3CA alteration status.

### Exclusion criteria

The exclusion criteria were (1) studies published in languages other than English, (2) replicate or overlapping publications, (3) analysis of only cancer cell lines, and (4) studies with a small sample size (≤20 patients).

### Quality assessment

The quality of the included studies was assessed independently by two researchers using the Newcastle–Ottawa Scale [22]. This scale is an eight-item instrument that assesses patient population and selection, study comparability, and outcomes. We considered a study high-quality if it was awarded six or more stars [23].

### Data extraction

The following items were extracted from the included studies: study title, name of the first author, publication year, region, PIK3CA alteration type, detection method, and clinicopathological characteristics (i.e., gender, TNM stage, and tumor differentiation). The HRs, ORs, and 95% CIs for the association of PIK3CA status with OS and clinicopathological features were collected from the studies. If HRs or ORs were not directly reported, survival data were calculated using the methods described by Parmar et al. [33] and Tierney et al. [34].

### Statistical analysis

Meta-analysis was performed using Stata version 12.0 software (StataCorp, College Station, TX, USA). A significant heterogeneity was observed when P < 0.05 or I^2^ > 50%, and a random-effects model was then applied. Otherwise, a fixed-effects model was used.

## SUPPLEMENTARY MATERIALS TABLE


