# Supplementary material for: Association between PIK3CA alteration and prognosis of gastric cancer patients: a meta-analysis
Source: Oncotarget. 2018 Jan 2;9(7):7651–9. doi: 10.18632/oncotarget.23871 (PMC5800933; doi:10.18632/oncotarget.23871)
Supplement: Supplementary file 1 [file oncotarget-09-7651-s001.pdf]

Association between PIK3CA alteration and prognosis of gastric cancer patients: a meta-analysis

SUPPLEMENTARY MATERIALS

Supplementary Table 1: The assessment of quality of each study according to the Newcastle-Ottawa Scale

Study	Selection(0-4)				Comparability		Outcome(0-3)			Total
	REC	Snec	AE	OINP	SCB	SCA	AO	FU	AFC	
S Barbi [11]	1	0	1	1	1	0	1	1	1	7
J Shi [12]	1	1	1	1	1	1	0	1	0	7
Y Sukawa [13]	1	0	1	1	1	0	1	0	0	5
AFC Okines [14]	1	1	1	1	1	1	1	1	1	9
ML Chong [15]	1	1	1	1	1	1	1	1	0	8
H Lee [16]	1	1	1	1	1	0	1	1	0	7
M Liang [17]	1	1	1	0	1	0	1	0	0	5
K Harada [18]	1	0	1	1	1	1	1	1	0	7
M Dong [19]	1	1	1	0	1	0	1	1	1	7
SH Jang [20]	1	1	1	0	1	1	1	1	0	7
JW Kim [21]	1	1	1	1	1	0	1	1	0	7

REC: representativeness of the exposed cohort; SNEC: selection of the nonexposed cohort; AE: ascertainment of exposure; OINP: outcome of interest not presented in the start of study; SCB: study controls for basic characteristics; SCA: study controls for additional factor; AO: assessment of outcome; FU: follow-up; AFC: adequacy of follow up.
